# Trust in independent community pharmacies: Do employee-related factors matter?

**DOI:** 10.4102/hsag.v25i0.1344

**Published:** 2020-07-02

**Authors:** Edwin Theron, Almeri Pelser

**Affiliations:** 1 Department of Business Management, Faculty of Economic and Management Sciences, Stellenbosch University, Stellenbosch, South Africa

**Keywords:** Independent community pharmacies, trust, employee-related factors, antecedents, management

## Abstract

**Background:**

Offering quality healthcare services in South Africa’s remote areas remains a challenge. Pharmacies, and independent community pharmacies (ICPs) in particular, can play a vital role in providing access to pharmaceutical products and services in these areas.

**Aim:**

Part of the success of ICPs is the role that their employees play in building trusting relationships with pharmacy clients. It is against this background that this article investigates key employee-related factors that contribute towards building affective, calculative and contractual trust when pharmacy clients are serviced.

**Setting:**

Clients of a specific ICP group participated in the study. The selected ICP group, which manages eight pharmacies across the Western Cape Province, has between 8000 and 41 000 active client profiles per pharmacy.

**Methods:**

All 299 respondents who participated in the study were personally interviewed. Statistical analyses were done through Statistica, and structural equation modelling (SEM) with partial least squares (PLS) was used to assess both the measurement and the structural model.

**Results:**

Although a number of significant relationships were confirmed, the importance of especially familiarity is highlighted when trust is managed in a pharmacy client-employee relationship.

**Conclusion:**

Given their geographical location, ICPs are ideally situated to provide access to healthcare services in the more remote areas of South Africa. By focusing on managing trust, ICPs can ensure a more constructive experience to their clients.

## Introduction and background

The South African healthcare industry faces a myriad challenges, of which the delivery of quality healthcare to all South Africans is arguably on the forefront. Although various strategies have been put in place to address this key challenge, the South African healthcare industry is undergoing sweeping changes in the policy and regulatory environment, such as the promulgation of the National Health Insurance (NHI) Bill and the Medical Schemes Amendment Bill. The pharmaceutical industry will also be significantly affected by these changes, especially as decisions still need to be taken about how medication will be acquired and how it will be distributed in future. Despite these challenges, it is evident that the role of pharmacists in improving patients’ quality of life is becoming increasingly important (Cheng et al. 2013). The independent community pharmacy (ICP), in particular, has always been crucial in providing access to pharmaceutical products and services to the broader population.

In addition, the global healthcare industry is characterised by distrust, as illustrated by the reputable Edelman Trust Barometer. For example, the Barometer found that trust in the healthcare industry has decreased among 17 of the 28 global markets measured (Edelman Trust Barometer: Trust in Healthcare 2018). Given the challenges that the healthcare industry faces along with the decrease in trust levels in the industry, it is not surprising that the pharmaceutical industry is rethinking its interest and approach towards relationship marketing practices (Clark, Vorhies & Bentley 2011).

Against this background, this article adopts a renewed approach about the management of trust among ICPs, by focusing on the important role that pharmacy employees can play in ensuring client trust. This objective is also suitable given the realisation that interactions between employees and customers have a significant impact on relational outcomes (Trainor et al. 2014).

### Trust

Trust does not only form the basis of all human interactions, but it is also one of the most complex constructs that governs behaviour (Hawlitschek, Teubner & Weinhardt 2016). In defining the concept of trust, Rousseau et al. (1998:395) argue that trust is ‘a psychological state comprising the intention to accept vulnerability based upon positive expectations of the intentions or behavior of another’.

The multidimensional nature of trust is illustrated by the fact that the concept consists of an affective (Punyatoya 2018), a cognitive (Terres, Dos Santos & Basso 2015) and a contractual component (Krishnan, Geyskens & Steenkamp 2016). Affective trust refers to the confidence one places in another on the basis of feelings generated by the level of care and concern the other party demonstrates (Johnson & Grayson 2005). As this type of trust is embedded in emotions, it tends to deepen over time as the parties make a mutual, emotional commitment to the relationship (Erdem & Ozen 2003). Cognitive-based trust implies that individuals seek rational reasons to trust another party (Erdem & Ozen 2003). High-consequence exchanges are generally complex situations that force customers to make an extra cognitive effort to evaluate their decisions and alternatives carefully (Terres et al. 2015). Contractual trust is found in a mutual understanding between partners to adhere to a specified agreement, which means that this type of trust is strongly related to moral standards of honesty and trustworthiness (Sako 2006).

### Trust in healthcare services

Trust is not only viewed as a central construct in healthcare context relationships, but it is also a crucial element through which health outcomes are reached (Hall et al. 2001). In terms of vulnerability, Gidman, Ward and McGregor (2012) argue that vulnerability arises when users of health care services are ill and require care in an environment of specialist knowledge.

**FIGURE 1:**
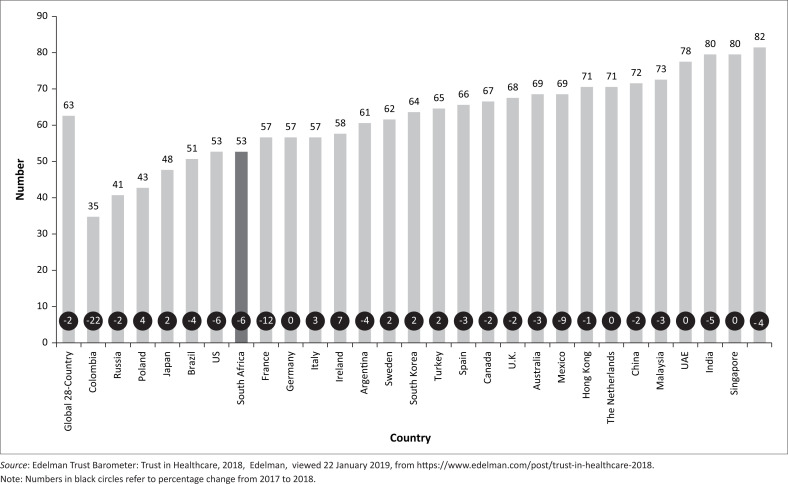
Trust in the global healthcare industry.

The downward spiral in global healthcare trust levels is portrayed in Figure 1.

Compared to the global average of 63%, trust in the South African healthcare industry has decreased from 59% to 53% from 2017 to 2018 (see Figure 1). Of the 28 countries analysed, South Africa is not only in the seventh worst position, but the 6% drop in the country from 2017 to 2018 is also significantly higher than the average drop of only 2%. More specifically, it was found that trust in the pharmaceutical industry amounts to only 55%.

Healthcare services are often characterised by uncertainty, risk and interdependence (Hosseini & Behaboudi 2017). Since one of the purposes of trust is to reduce uncertainty and risk (Chiu et al. 2018), it is inferred that trust is also a key building block of successful relationships in healthcare services.

Trust is achieved by means of a central route of information processing that coincides with a client’s high level of involvement when visiting a pharmacy (Perepelkin & Zhang 2011). Therefore, firms have a direct impact on customers’ health, with regard to building of trust as an important objective (Meijboom, Visak & Brom 2006). Berry and Bendapudi (2007) add that healthcare services are highly reliant on trust as the patient has to surrender completely to the healthcare provider (seller) so that the latter can solve their problem and address their needs. From a service provider’s perspective, including healthcare, trust results in favourable outcomes such as customer loyalty, repurchase intentions and customer satisfaction (Kantsperger & Kunz 2010).

### Employee-related factors and trust

A frontline employee refers to a service worker who personally interacts with a customer in a retail or a service environment (Sirianni et al. 2009). Frontline employees are a major source of communication, linking customers to firms and, consequently, influencing customers’ intention to maintain relations with the particular firm (Guenzi & Georges 2010). These employees not only make satisfactory exchanges possible, but they also have the ability to drive customer trust (Chen & Quester 2015). Furthermore, the building of trust between customers and frontline employees has been found to be one of the most cited actions in establishing long-term customer–firm relationships (Gremler, Gwinner & Brown 2001). However, despite the importance of trust, research on the employee-related antecedents of trust remains limited, especially from the perspective of ICPs. This article proposes that five key employee related antecedents contribute to trust in the pharmaceutical industry: expertise, familiarity, communication skills, likeability and customer orientation.

Employee expertise refers to the knowledge, experience and general competencies of an employee (Crosby, Evans & Cowles 1990). Competency, which is often used interchangeably with expertise, refers to employees’ performance and the way in which they manage their tasks as well as the tactics they use to fulfil customers’ needs (Tohidinia & Haghighi 2011). Customers’ feelings of uncertainty and the perception of risk during service delivery may be reduced through employees’ knowledge, technical competence and ability to answer specific questions (Guenzi & Georges 2010). Thus, employees whom customers perceive as possessing a high level of expertise provide customers with added value, which may lead to stronger and longer lasting relational ties between them (Liu & Leach 2001). Based on the literature review, the following hypotheses were stated:

**H**_**1a, b, c**_: A positive relationship exists between customers’ perception of employees’ expertise and (1) affective trust, (2) cognitive trust, and (3) contractual trust.

Familiarity refers to a customer’s perception that an employee recognises the customer and knows what the customer’s specific services needs are (Gremler et al. 2001). Pressey and Mathews (2000) assert that the level of contact between the customer and employee is central to establishing mutually committed relationships. Gremler et al. (2001) contend that a customer’s trust is more likely to develop when an employee and customer are well known to each other because of repeated encounters. In addition, repeated interactions between customers and employees can assist customers in the process of assessing the employee’s credibility and benevolence (Kantsperger & Kunz 2010), which are key components of trust. The following three hypotheses were therefore formulated:

**H**_**2a, b, c**_: A positive relationship exists between customers’ perception of familiarity with employees and (1) affective trust, (2) cognitive trust, and (3) contractual trust.

Communication is defined as the formal and informal information sharing of meaningful and timely information between relevant parties (Theron & Terblanche 2010). Employees act as the primary source of communication, especially in retail market situations (Sharma et al. 1999). Based on a systematic review of the literature, Phelps and Campbell (2012) confirmed that communication is a key driver of trust in any relationship. It was therefore hypothesised that:

**H**_**3a, b, c**_: There is a positive relationship between customers’ perception of employees’ communication skills and (1) affective trust, (2) cognitive trust, and (3) contractual trust.

Likeability is described as the extent to which an employee is perceived to be friendly, courteous and pleasant (Guenzi & Georges 2010). Perceived likeability deals with personality-related factors such as the degree to which an employee has an empathetic attitude, behaves politely and shares similar tastes, preferences, values and beliefs, status, appearance, lifestyle and personality characteristics with a customer (Coulter & Coulter 2003). In addition, it was found that employee characteristics and behaviour play an important role in the development of trust and contribute to how customers perceive a firm (Ivens & Schaarschmidt 2015). Based on this literature review, the following hypotheses were formulated:

**H**_**4a, b, c**_: There is a positive relationship between customers’ perception of employees’ likeability and (1) affective trust, (2) cognitive trust, and (3) contractual trust.

According to Kemp, Jillapalli and Becerra (2014), it is crucial for employees to demonstrate customer-oriented behaviour as it contributes to building trust. Luo, Hsu and Liu (2008) believe that customer orientation is a set of beliefs that puts the customer’s interest first, while not excluding those of all other stakeholders. Customer orientation also suggests that employees alter and customise services to suit customers’ specific needs (Coulter & Coulter 2003). Newell et al. (2011) argue that customer-oriented behaviour plays a vital role in influencing buyer perceptions of trust during interactions. Since the central focus of customer-orientated employees is the well-being of customers, the latter will be more inclined to place trust in businesses which they feel care about their well-being (Van Esterik-Plasmeijer & Van Raaij 2017). The following hypotheses were consequently stated:

**H**_**5a, b, c**_: There is a positive relationship between customers’ perception of employees’ customer orientation and (1) affective trust, (2) cognitive trust, and (3) contractual trust.

## Methods

### Context

The South African private pharmacy industry is primarily divided into two groups: corporate chains, which are large retail pharmacy chains under corporate ownerships, and independent community pharmacies (ICPs), which are either individually owned and/or managed or which are part of a smaller group. This study specifically focused on ICPs. Independent community pharmacies play a unique role in offering services in geographical areas where people do not necessarily have access to larger corporate pharmacy chains. The uniqueness of ICPs lies in the fact that these pharmacies can offer more than a mere commercial transaction only, by focusing on a more personal experience with their clients (Perepelkin & Zhang 2014). These authors argue that clients’ trust in a pharmacy is largely derived from their perceptions of the pharmacy’s employees per se rather than the actual products the employees sell. This notion implies that pharmacies have to redefine the roles of their employees (Smith et al. 2018).

### Measurement instrument

A 40-item questionnaire was used to measure the perceptions of ICP clients. The items were mainly sourced from Guenzi and Georges (2010), Johnson and Grayson (2005), Miyamoto and Rexha (2004), Terres et al. (2015) and Tohidinia and Haghighi (2011). Five items were used to measure the five independent and the three dependent variables. All questions were assessed using a 7-point Likert scale.

### Sampling

Clients of a preselected ICP group that manages eight pharmacies across the Western Cape Province participated in the study. This ICP group has between 8000 and 41 000 active customer profiles per pharmacy. In order to obtain a representative number of respondents, it was decided to obtain 50 questionnaires from each pharmacy, envisaging 400 completed responses.

### Data collection

Structured interviews were conducted with clients waiting to be served by a pharmacist. Those customers visiting the pharmacies to purchase non-medical supplies (such as haircare products), were therefore excluded. Data collection occurred over a 4-week period.

### Data analysis

All analyses were done using Statistica, an advanced software package originally developed by StatSoft in the USA. The reliability of the instrument was assessed by determining the Cronbach alpha coefficients for each variable, and structural equation modelling (SEM) with partial least squares (PLS) was used to assess both the measurement and the structural models.

### Ethical consideration

Ethical clearance for the study was obtained from both the institution (the Research Ethics Committee at Stellenbosch University, approval number SU-HSD-000435) to which the authors are affiliated and the managing director of the ICP group participating in the study. In addition, written consent was obtained from each participant.

## Empirical results

A pilot study was conducted to establish the reliability of the measurement instrument. The Cronbach alpha values ranged between 0.897 and 0.986, providing evidence of sufficient reliability. In the main study, a total of 299 respondents participated. Of these 299 respondents, 60.2% were female and 39.8% were male. This uneven gender distribution can be ascribed to the fact that, as women are often known to fulfil the role of primary caretaker in more traditional households, they are the ones to visit a pharmacy when medication is required. Once again, the reliability scores were determined, and the Cronbach alpha scores ranged between 0.930 and 0.968, illustrating sufficient levels of internal reliability.

**FIGURE 2:**
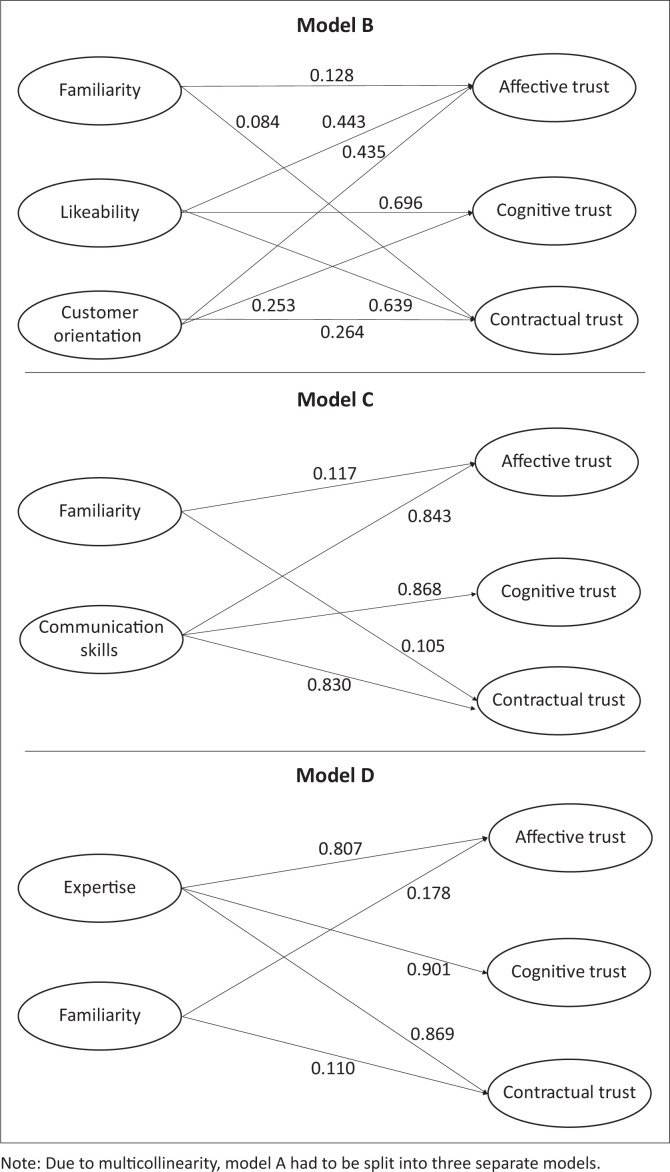
Structural Models B, C and D.

Inferential statistics were obtained from employing PLS-SEM, allowing for the assessment of the measurement (outer) and the structural (inner) models (Hair et al. 2014). Once the conceptual model (Figure 2) was assessed, it became evident that multicollinearity might be a concern. This concern was addressed by constructing three smaller models, based on the exclusion of the highly correlated independent variables. In order to achieve valid and more robust results, the same statistical techniques were applied to all three models. The initial conceptual model was labelled Model A, and the three additional models were labelled Models B, C and D. Model B hypothesised familiarity, likeability and customer orientation (the three independent variables) relative to affective, cognitive and contractual trust (the three dependent variables). Model C had familiarity and communication skills as independent variables, whereas expertise and familiarity were the two independent variables in the case of Model D.

### Measurement model assessment

Reliability and validity tests were executed by employing partial least squares (PLS) in order to assess the measurement model. Firstly, four outer models were assessed by evaluating the internal consistency. Thereafter, the convergent and discriminant validity of these frameworks were also evaluated. Internal consistency signals that measure behave as expected, which proves that they are related to the same construct. Table 1 indicates the calculated composite reliability, the average variance extracted (AVE) and outer loadings for each variable.

**TABLE 1:** Assessment of the measurement model: Model A.

Item	Variable	Composite reliability	AVE	Outer loading
AFF1	Affective trust	0.956	0.813	0.882
AFF2				0.928
AFF3				0.850
AFF4				0.919
AFF5				0.928
COG1	Cognitive trust	0.976	0.889	0.900
COG2				0.966
COG3				0.956
COG4				0.945
COG5				0.946
CON1	Contractual trust	0.970	0.865	0.929
CON2				0.928
CON3				0.939
CON4				0.927
CON5				0.926
EXP1	Expertise	0.973	0.877	0.919
EXP2				0.957
EXP3				0.942
EXP4				0.943
EXP5				0.922
CUS1	Customer orientation	0.960	0.828	0.926
CUS2				0.894
CUS3				0.896
CUS4				0.919
CUS5				0.913
FAM1	Familiarity	0.947	0.781	0.912
FAM2				0.883
FAM3				0.823
FAM4				0.884
FAM5				0.914
LIK1	Likeability	0.962	0.837	0.946
LIK2				0.939
LIK3				0.912
LIK4				0.934
LIK5				0.839
COM1	Communication skills	0.968	0.860	0.928
COM2				0.938
COM3				0.943
COM4				0.906
COM5				0.922

AVE, Average variance extracted; AFF, Affective trust; COG, Cognitive trust; CON, Contractual trust; CUS, Customer orientation; LIK, Likeability; FAM, Familiarity; COM, Communication skills; EXP, Expertise.

Table 1 demonstrates that all constructs provide evidence of construct reliability as their values are all above the 0.70 threshold. In terms of convergent validity, outer loadings were inspected. The AVE values and outer loadings for Models A to D changed only marginally after each model was assessed individually. Therefore, it was decided not to report each one of these values, but only the reliability statistics for the initial full model, Model A. Convergent validity was therefore confirmed. Discriminant validity was assessed through the Heterotrait-Monotrait (HTMT) ratio of correlations, and the results appear in Table 2.

**TABLE 2:** Heterotrait-Monotrait ratio: Model A.

Variable	Original sample	Lower limit 2.5%	Upper limit 97.5%	Discriminant validity
COG→AFF	0.990	0.975	1.002	No
CON→AFF	0.992	0.974	1.009	No
CON→COG	0.996	0.983	1.008	No
CUS→AFF	0.972	0.939	0.999	Yes
CUS→COG	0.930	0.855	0.981	Yes
CUS→CON	0.931	0.865	0.981	Yes
LIK→AFF	0.975	0.944	0.995	Yes
LIK→COG	0.982	0.956	0.996	Yes
LIK→CON	0.977	0.949	0.994	Yes
LIK→CUS	0.932	0.859	0.976	Yes
COM→AFF	0.981	0.954	0.998	Yes
COM→COG	0.944	0.901	0.971	Yes
COM→CON	0.947	0.905	0.977	Yes
COM→CUS	0.978	0.951	1.00	No
COM→LIK	0.952	0.910	0.980	Yes
EXP→AFF	0.983	0.963	0.997	Yes
EXP→COG	0.984	0.961	0.997	Yes
EXP→CON	0.986	0.966	0.999	Yes
EXP→CUS	0.956	0.897	0.996	Yes
EXP→LIK	0.974	0.945	0.992	Yes
EXP→COM	0.971	0.951	0.987	Yes
FAM→AFF	0.809	0.740	0.868	Yes
FAM→COG	0.753	0.677	0.821	Yes
FAM→CON	0.777	0.707	0.838	Yes
FAM→CUS	0.757	0.672	0.829	Yes
FAM→LIK	0.777	0.692	0.844	Yes
FAM→COM	0.809	0.743	0.868	Yes
FAM→EXP	0.760	0.678	0.827	Yes

AFF, Affective trust; COG, cognitive trust; CON, contractual trust; CUS, customer orientation; LIK, likeability; FAM, familiarity; COM, communication skills; EXP, expertise.

The HTMT ratio confirmed the discriminant validity of the majority of the reflective constructs as most of these ratios were below 1 (see Table 2). However, in all four models the dependent variables (affective trust, cognitive trust and contractual trust) correlated too highly with each other’s scales, implying that these constructs were not completely distinct from each other by empirical standards. The same correlation occurred between communication skills and customer orientation for Model A as shown in Table 3, indicating that these two constructs captured some phenomena represented by both. By excluding either one of, or both communication skills and customer orientation, discriminant validity for all the independent variables were established with an HTMT ratio below one.

**TABLE 3:** Collinearity statistics (VIF): Model A.

Variable	AFF	COG	CON	CUS	LIK	COM	EXP	FAM
**Model A**								
AFF	-	-	-	-	-	-	-	-
COG	-	-	-	-	-	-	-	-
CON	-	-	-	-	-	-	-	-
CUS	8.84	8.84	8.84	-	-	-	-	-
LIK	8.69	8.69	8.69	-	-	-	-	-
COM	13.15	13.15	13.15	-	-	-	-	-
EXP	12.45	12.45	12.45	-	-	-	-	-
FAM	2.55	2.55	2.55	-	-	-	-	-

AFF, Affective trust; COG, cognitive trust; CON, contractual trust; CUS, customer orientation; LIK, likeability; COM, communication skills; EXP, expertise; FAM, familiarity.

### Structural model assessment

Partial least squares was used to assess the structural model of Models A to D, which shows the relationships (paths) between the latent constructs. Three assessments were conducted, namely collinearity concerns in terms of the structural model, the determination of *R*-square (*R*^2^), and the significance and relevance of the structural model relationships.

Table 3 provides the collinearity statistics of the four separate models because of collinearity issues that surfaced after assessing the initial full model (Model A). This threat of multicollinearity then justified the assessment of the three additional models in order to explore whether different combinations of independent variables produced lower VIF values to report valid results.

As indicated by Table 3, all VIF values pertaining to Model A exceeded the generally accepted level of 5, with the exception of familiarity. Therefore, it may be inferred that there is a correlation between the remaining variables. Familiarity yielded satisfactory results pertaining to reliability and discriminant validity. In comparison with all other variables, familiarity could easily be distinguished by the respondents as a distinct concept, whereas the remaining variables could have been perceived as more ambiguous by some respondents, resulting in high VIF values. As explained earlier, this concern was addressed by creating three separate models (labelled as Models B, C and D). The collinearity statistics for the three models appear in Table 4.

**TABLE 4:** Collinearity statistics (VIF): Models B, C and D.

Variable	AFF	COG	CON	CUS	LIK	COM	EXP	FAM
**Model B**								
AFF	-	-	-	-	-	-	-	-
COG	-	-	-	-	-	-	-	-
CON	-	-	-	-	-	-	-	-
CUS	4.889	4.889	4.889	-	-	-	-	-
LIK	5.137	5.137	5.137	-	-	-	-	-
FAM	2.321	2.321	2.321	-	-	-	-	-
**Model C**								
AFF	-	-	-	-	-	-	-	-
COG	-	-	-	-	-	-	-	-
CON	-	-	-	-	-	-	-	-
COM	2.501	2.501	2.501	-	-	-	-	-
FAM	2.501	2.501	2.501	-	-	-	-	-
**Model D**								
AFF	-	-	-	-	-	-	-	-
COG	-	-	-	-	-	-	-	-
CON	-	-	-	-	-	-	-	-
EXP	2.168	2.168	2.168	-	-	-	-	-
FAM	2.168	2.168	2.168	-	-	-	-	-

AFF, Affective trust; COG, cognitive trust; CON, contractual trust; CUS, customer orientation; LIK, likeability; FAM, familiarity; COM, communication skills; EXP, expertise.

Model B was created by excluding the variables that proved to have the highest VIF values, namely communication skills and expertise. As a result, the VIF values in Model B decreased significantly. Although likeability still produced a VIF value greater than five, it was only marginally so. Therefore, as likeability was only moderately correlated with the remaining variables, there was little cause for concern. Consequently, likeability and customer orientation were excluded from Model C. However, communication skills was reintroduced into the model and, ultimately, the two remaining independent variables produced satisfactory VIF values, as can be seen in Table 4. In addition, Model D was also created to assess the collinearity of the model if expertise were also to be reintroduced, but replaced communication skills in Model C. The outcome also proved to be satisfactory since the two remaining independent variables produced the lowest VIF values in comparison with the other three models.

#### Assessment of coefficient of determination (*R*^2^)

An assessment of the coefficient of determination (*R*^2^) indicates the percentage of variance that is explained by the independent variables of a conceptual model. In the present study, the *R*^2^ results reveal that for Model B, the values were 0.908, 0.878 and 0.894 (for affective, cognitive and contractual trust respectively). For Model C, these values were 0.906, 0.834 and 0.907, whereas Model D revealed values of 0.894, 0.835 and 0.907. In all cases, the results showed that a substantial amount of the variance in affective, cognitive and contractual trust could be explained among all three models.

#### Assessment of path coefficients

In order to assess the various paths between the different constructs, the standardised regression weights of these models were examined. The hypotheses were assessed in Models B, C and D only, because of multicollinearity issues with model A. The results for these three models are shown in Figure 2.

The first structural model to be assessed was Model B. In terms of affective trust, all three of the independent variables produced *p*-values below the significant level of 0.05, implying that these three variables are positively and significantly related to affective trust. In addition, likeability was identified as the variable with the biggest effect on affective trust with a path coefficient of 0.443. Both customer orientation and likeability were found to be positively and significantly related to cognitive trust. Familiarity, however, was not significantly related to cognitive trust. Finally, contractual trust showed to be positively and significantly related to all three employee-related dimensions.

Structural Model C (shown in Figure 2) had familiarity and communication skills as independent variables. From Figure 2 it is evident that both familiarity and communication skills were significantly related to affective trust. Only communication skills was found to be positively related to the dependent variable cognitive trust. Also, both familiarity and communication skills were positively and significantly related to contractual trust.

The final structural model was Model D, which had expertise and familiarity as independent variables. Once again, cognitive trust demonstrated an insignificant relationship with familiarity (*p* = 0.1, *p* > 0.05). However, expertise was found to be positively and significantly related to cognitive trust (*p* < 0.01) with a great effect size also established between the two variables, as indicated by a path coefficient of 0.901. Finally, contractual trust also showed to be positively and significantly influenced by both the employee-related dimensions, expertise and familiarity (*p* < 0.05). Expertise had the greatest effect on contractual trust with a path coefficient of 0.869 as opposed to familiarity, which produced a coefficient of 0.11.

## Hypotheses test results

Table 5 provides a summary of the results of the hypotheses assessed.

**TABLE 5:** Summary of the empirical results.

Number	Hypothesis description	Finding
H_1a_	There is a positive relationship between customers’ perception of employees’ expertise and affective trust	Partially supported in Model D
H_2a_	There is a positive relationship between customers’ perception of familiarity with employees and affective trust	Supported in Models B, C & D
H_3a_	There is a positive relationship between customers’ perception of employees’ communication skills and affective trust	Partially supported in Model C
H_4a_	There is a positive relationship between customers’ perception of employees’ likeability and affective trust	Partially supported in Model B
H_5a_	There is a positive relationship between customers’ perception of employees’ customer orientation and affective trust	Partially supported in Model B
H_1b_	There is a positive relationship between customers’ perception of employees’ expertise and cognitive trust	Partially supported in Model D
H_2b_	There is a positive relationship between customers’ perception of familiarity with employees and cognitive trust	Not supported
H_3b_	There is a positive relationship between customers’ perception of employees’ communication skills and cognitive trust	Partially supported in Model C
H_4b_	There is a positive relationship between customers’ perception of employees’ likeability and cognitive trust	Partially supported in model B
H_5b_	There is a positive relationship between customers’ perception of employees’ customer orientation and cognitive trust	Partially supported in Model B
H_1c_	There is a positive relationship between customers’ perception of employees’ expertise and contractual trust	Partially supported in Model D
H_2c_	There is a positive relationship between customers’ perception of familiarity with employees and contractual trust	Supported in Models B, C & D
H_3c_	There is a positive relationship between customers’ perception of employees’ communication skills and contractual trust	Partially supported in Model C
H_4c_	There is a positive relationship between customers’ perception of employees’ likeability and contractual trust	Partially supported in Model B
H_5c_	There is a positive relationship between customers’ perception of employees’ customer orientation and contractual trust	Partially supported in Model B

## Theoretical contribution

By focusing on the management of trust when client–employee relationships are managed, the study contributes to a relatively under researched topic. Consistent with the viewpoint of Liu and Leach (2001), this study emphasises that when pharmacy employees are perceived as having high levels of expertise, it results in stronger relational ties between the pharmacy employees and their clients. The results confirm that the technical capabilities of employees not only drive relational outcomes (Hennig-Thurau 2004), but that these also enhance employees’ credibility and trustworthiness (Johnson & Grayson 2005). Thus, when pharmacy clients perceive that an employee has the necessary skills and knowledge to provide an acceptable service, it serves as an affective signal that instils trust based on emotion. Likewise, expertise significantly influences a pharmacy client’s confidence or willingness to trust the service provider’s reliability and honourability, given this dimension’s positive relationship with cognitive and contractual trust.

As far as familiarity is concerned, the study found that pharmacy clients are more likely to develop trust in an employee when employee and client are well known to each other. This finding concurs with that of Gremler et al. (2001). Furthermore, the results are in line with those of Kantsperger and Kunz (2010), that repeat interactions between customers and employees assist customers in assessing credibility and benevolence. Thus, this finding shows that familiarity contributes to a pharmacy client’s belief that the service provider will keep their promise and honour an agreement with the client.

It is almost generally assumed that the exchange of information shared between parties has the ability to build stronger relationships. For example, Sharifi and Esfidani (2014) found that frequent information exchange is an important building block of trust in the development of long-term relationships between customers and firms. The findings of this study concur with those of Sharifi and Esfidani (2014) and of Tohidinia and Haghighi (2011) that mutual communication leads to increased confidence in relationships.

Likeability was found to be positively associated with affective, cognitive and contractual trust, which is in agreement with an earlier finding by Coulter and Coulter (2003). Furthermore, the results confirm the expectation that interpersonal liking drives different types of trust in a pharmacy setting; this finding concurs with the view of (Hennig-Thurau 2004). Ultimately, the implication of this finding is that when pharmacy clients are able to identify with employees on a personal level and on shared similarities, it induces rational, emotional and moral connections to the pharmacy.

Prior research showing that customer orientation positively contributes to relationship quality (Kemp et al. 2014) is consistent with the results of this study. The findings of this study support the hypotheses that pharmacy clients’ perceptions of employees’ customer orientation are positively related to affective, cognitive and contractual trust. Thus, understanding pharmacy clients’ needs and expectations and customising services to suit those specific needs significantly influence clients’ expectations that the service provider will keep their promise (Miyamoto & Rexha 2004).

## Managerial implications

Independent community pharmacies (ICPs) are often poorly resourced with fewer diverse product ranges than leading pharmacy groups or chains. Therefore, ICPs increasingly have to find innovative ways to differentiate themselves. This article argues that this differentiation could be based on managing clients’ trust in pharmacy employees. In general, this article postulates that ICPs should rather focus on employeerelated (human resources) practices as opposed to expensive marketing efforts.

Pharmacy clients’ perceptions of employees’ expertise was indicated as influencing the clients’ affective, cognitive and contractual trust in the pharmacy, which refers to feelings of a deep emotional, rational and moral connection with the service provider. Therefore, aspects pertaining to the enhancement of employees’ skills and knowledge should be managed to such an extent that employees are able to deliver service with confidence. In addition, employee training should be viewed as a collective process where, for example, pharmaceutical representatives accept responsibility for training on new products.

In terms of employees’ communication skills, the pharmacies’ focus should be on communicative abilities, such as proper language use, communicating in a warm and personal manner, as well as continuous emphasis on non-verbal communication (such as attitude and body language). A pharmacy remains a professional environment, and clients should be treated accordingly. Part of this approach is that pharmacy employees should be client-orientated, which means that pharmacies should be attuned to their clients’ needs.

Likeability was found to be a further contributor to all three types of trust. Once pharmacy employees are perceived as likeable, they significantly add to the client–employee relationship. Pharmacy managers who set an example of courteous behaviour towards their clients will encourage other employees to follow suit.

Familiarity was found to be the most important antecedent of trust in this study. Familiarity could be fostered by focusing on maintaining a high employee retention rate, thereby minimising high employee turnover rates. From a customer’s perspective, familiarity is enhanced through customer retention strategies, such as reward programmes. Reward programmes have the added benefit that pharmacies could identify their most valuable clients.

It should be kept in mind that in the ICP context, trust is a multidimensional construct that should be managed on three distinct levels, namely affective, cognitive and contractual levels. Independent community pharmacies should therefore be cautious not to develop an almost myopic view of trust by disregarding the individual components of the concept.

## Limitations and future research

After assessing the measurement model, it was found that the three types of trust were not completely discriminant from each other. The assumption can be made that South Africa’s political history and cultural differences could have contributed to the result, as different results in discriminant validity could be found if the study were to be conducted in a different geographical and political context. However, research on how to deal with this challenge in this particular context is limited.

Furthermore, despite the fact that the study made use of well-established scales, relatively high levels of multicollinearity among the independent variables were experienced. One should therefore be cautious to apply scales that were developed in developed markets to emerging markets such as South Africa.

Although it was not the objective of this study, it would have been interesting to assess whether the cultural differences in South Africa could have influenced the results of the study.

## Conclusion

This study highlighted the importance of a number of antecedents when trust in ICPs is fostered and managed. Trust in a client–pharmacist relationship is developed and managed on a personal or individual level, which re-emphasises the important role that pharmacy employees play in ensuring trust. In broader terms, the article encourages ICPs to rethink their current trust levels.

Building or regaining trust levels in ICPs could have significantly positive effects, such as offering ICPs a tool to gain a competitive advantage amidst fierce competition in the healthcare services industry. However, from a broader perspective, possibly the most significant benefit of focusing on trust is the contribution that ICPs can make towards the overall objective of providing access to healthcare services to the broader South African population.
